# Effect of Mixed Transplantation of Autologous and Allogeneic Microskin Grafts on Wound Healing in a Rat Model of Acute Skin Defect

**DOI:** 10.1371/journal.pone.0085672

**Published:** 2014-01-21

**Authors:** Heng Lin, Yanni Yang, Yong Wang, Lihua Wang, Xin Zhou, Jing Liu, Daizhi Peng

**Affiliations:** 1 Institute of Burn Research, Southwest Hospital, Third Military Medical University, Chongqing, China; 2 Tissue Engineering Research Unit, State Key Laboratory of Trauma, Burns and Combined Injury, Third Military Medical University, Chongqing, China; University Hospital Hamburg-Eppendorf, Germany

## Abstract

The treatment of extensive thermal injuries with insufficient autologous skin remains a great challenge to burn surgeons. In this study, we investigated the influence of the ratio of autologous and allogeneic tissue in mixed microskin grafts on wound healing in order to develop an effective method for using limited donor skin to cover a large open wound. Four different mixtures were tested: autologous microskin at an area expansion ratio of 10∶1 with allogeneic microskin at an area expansion ratio of 10∶1 or 10∶3 and autologous microskin at an expansion ratio of 20∶1 with allogeneic microskin at an expansion ratio of 20∶3 or 20∶6. Wound healing, wound contraction, and integrin β1 expression were measured. Mixed microskin grafting facilitated wound healing substantially. The mixture of autologous microskin at an expansion ratio of 10∶1 with the same amount of allogeneic microskin achieved the most satisfactory wound healing among the 4 tested mixtures. Histological examination revealed the presence of obviously thickened epidermis and ectopic integrin β1 expression. Keratinocytes expressing integrin β1 were scattered in the suprabasal layer. Higher levels of integrin β1 expression were associated with faster wound healing, implying that ectopic expression of integrin β1 in keratinocytes may play a pivotal role in wound healing. In conclusion, this study proves that this new skin grafting technique may improve wound healing.

## Introduction

Early closure of open wounds in the treatment of extensive thermal injury with insufficient autologous skin has been a great challenge to burn surgeons. The available methods of skin grafting remain limited to techniques including intermingled skin grafting using autologous skin islets inlaid in an allogeneic skin sheet [1], autologous microskin transplantation overlaid with viable allograft or xenograft skin [2], and in vitro cultured keratinocyte grafts [3].

Intermingled skin grafting has been used clinically for more than 40 years and represented a significant breakthrough, increasing the survival of patients with full-thickness skin burns over 50% of the total body surface area (TBSA). Its main advantages are that the autologous keratinocytes can induce local tolerance to allograft or xenograft tissue [1], [4], which delays graft rejection, and that the autologous epithelium infiltrates quickly between the epidermis and dermis of the allograft or xenograft skin. However, this type of skin grafting requires 2-stage surgery, first burn eschar excision and coverage of the defect with allogeneic or xenogeneic skin followed by insertion of small autologous skin sheets into the allogeneic skin 3–6 days after the first surgery. This approach not only imposes the stress of surgery twice within a short time but also results in extensive scarring after wound healing.

Zhang et al. [2] developed a technique for treating extensive burns with limited areas of autologous donor skin in which autologous microskin grafts are overlaid on a viable allograft; this effective method has been applied widely in China. The keratinocytes from the autologous microskin can proliferate actively and spread rapidly to resurface a wound ≥10 times the area of the donor site without a complex and expensive culture process or a prolonged period of cultivation. Nevertheless, primary healing accounts for only 54.9% of the area and secondary healing for 40.1%, with little cyst formation and obvious scarring following wound closure [5].

Cultured keratinocyte grafting is another approach to early closure of an open wound without sufficient donor skin [3]. However, its complexity, the long period required for cultivation, and the inconsistent success after transplantation lead to limited application in burn patients. Mixed cultivation of autologous and allogeneic keratinocytes can shorten the cultivation time and decrease the number of autologous keratinocytes needed [3], [6]. A method of increasing the expansion ratio further while achieving rapid and qualified wound healing would enable surgeons to manage extensive open wounds without worrying that the donor skin might be insufficient.

In this study, we resurfaced a full-thickness skin defect in rats with a mixture of autologous and allogeneic microskin grafts and investigated the effect of the ratio of the skin types on the wound healing and wound contraction rates. The histological appearance and integrin β1 expression of the healed wound were examined by performing hematoxylin/eosin and immunohistochemical staining to explore the primary mechanism of wound healing after application of this technique. We found that this new approach can promote wound healing and increase ectopic expression of integrin β1 in the healed epidermis. Therefore, this study provides strong evidence for the use of this new method of skin grafting to improve wound healing despite an extremely limited donor site.

## Materials and Methods

### Ethics statement

The animal studies were approved by the Animal Care and Use Committee of The Third Military Medical University, and all of the protocols were approved by the Ethics Committee of Southwest Hospital, Third Military Medical University, Chongqing, China.

### Animals and experimental groups

In this study, male Wistar rats weighing 222–260 g served as allogeneic microskin donors and female Sprague-Dawley (SD) rats weighing 203–265 g were recipients. These rats were provided by the Animal Center of The Third Military Medical University. The animals were housed in wire-bottomed, wire-lidded cages, allowed access to food and water ad libitum, and acclimated for 1 week in a temperature-controlled room with a normal 12-h light/dark cycle prior to the experiments.

### Experiment A

Female SD rats (N = 40) were divided into 4 groups that received different mixtures of autologous and allogeneic microskin: group I (n = 10, allogeneic donor microskin at an area expansion ratio of 10∶3 without autologous microskin), group II (n = 10, autologous microskin at 10∶1), group III (n = 10, both autologous and allogeneic microskin at 10∶1 each), and group IV (n = 10, autologous and allogeneic microskin at 10∶1 and 10∶3, respectively).

### Experiment B

Female SD rats (N = 30) were divided into 3 groups that received different mixtures of autologous and allogeneic microskin: group I (n = 10, autologous microskin only at an area expansion ratio of 20∶1), group II (n = 10, autologous and allogeneic microskin at 20∶1 and 20∶3, respectively), and group III (n = 10, autologous and allogeneic microskin at 20∶1 and 20∶6, respectively). The area expansion ratios of the different groups in experiments A and B are shown in detail in Table 1.

**Table 1:** Area expansion ratios in the different groups in Experiment A and Experiment B.

Experiment	Group	Number	Autologous microskin	Allogeneic microskin	Total microskin
Experiment A	Group I	10		10∶3	10∶3
	Group II	10	10∶1		10∶1
	Group III	10	10∶1	10∶1	10∶2
	Group IV	10	10∶1	10∶3	10∶4
Experiment B	Group I	10	20∶1		20∶1
	Group II	10	20∶1	20∶3	20∶4
	Group III	10	20∶1	20∶6	20∶7

### Full-thickness skin defect wound model and mixed microskin transplantation

**Figure 1:**
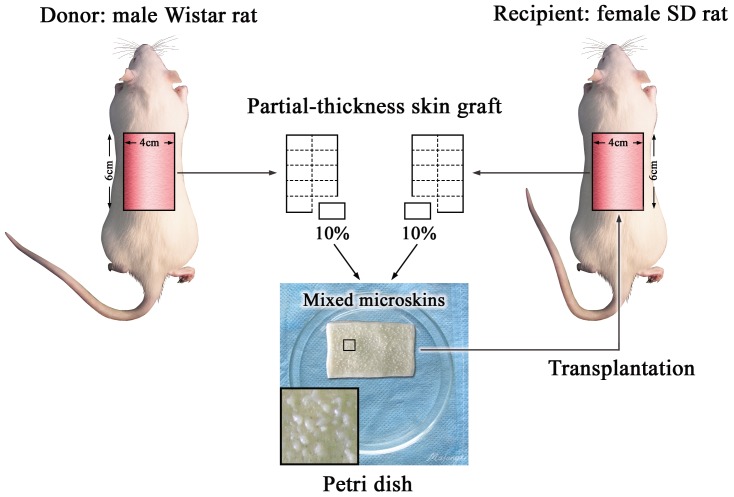
Schematic diagram of mixed microskin transplantation. The diagram shows the procedure of transplantation of mixed autologous and allogeneic microskin particles at identical area expansion ratios of 10∶1. The insert shows the even distribution of the mixed microskin particles on the paraffin gauze.

All rats were anesthetized by using intraperitoneal injection of sodium pentobarbital (45 mg/kg for Wistar rats and 40 mg/kg for SD rats). The dorsum was shaved and the exposed skin was washed gently with soap and room-temperature sterile water. A 4.0 cm×6.0 cm rectangular area along the dorsal midline was outlined by using a surgical skin marker and disinfected with povidone-iodine solution. A full-thickness skin defect 4.0 cm×6.0 cm in size was then created, leaving the underlying panniculus carnosus intact. Full-thickness skin grafts from both donors and recipients were trimmed by using a drum-type dermatome to yield 0.3-millimeter partial-thickness skin grafts (all of the epidermis and part of the dermis were preserved), which were weighed individually by using an electronic balance (Sartorius BP61, Germany). For example, when the expansion ratio of autologous skin was to be 10∶1, a 1/10 area of recipient partial-thickness graft (by weight) was harvested, cut into microskin by using fine surgical scissors, and preserved for later transplantation. Each microskin particle was less than 1 mm^3^ in volume. The microskin fragments were kept moist with sterile saline until transplantation onto the recipient wound. A piece of sterile liquid paraffin gauze slightly larger than the wound was used as an overlay. First, one side of the gauze was sutured to the wound. Then, the microskin fragments were spread as evenly as possible on the wound side of the paraffin gauze by using surgical forceps. Finally, the paraffin gauze was turned over to cover the wound and sutured on the other 3 sides. The individual orientations of the microskin particles were ignored. Bacterial prophylaxis was implemented immediately by applying chlorotetracycline ointment to the area around the wound and the outside of the gauze after transplantation. The wound was then dressed with several layers of sterile gauze on the outer surface. A cannular elastic bandage was used to keep the dressing in position. After recovery from anesthesia, each rat was housed in an individual cage and received a normal diet ad libitum. The procedure is shown in detail in Figure 1. The measures necessary for pain relief were carefully considered and applied to all animals before skin harvest from the donors and recipients, the microskin transplantation operation, and each postoperative dressing change for wound observation. Briefly, all rats were anesthetized by using intraperitoneal injection of sodium pentobarbital (45 mg/kg for Wistar rats and 40 mg/kg for SD rats) before skin harvest, surgery, and each dressing change. At the end of the study, the rats were euthanized by intraperitoneal injection of sodium pentobarbital (135 mg/kg for Wistar rats and 120 mg/kg for SD rats). Liquid paraffin gauze was used to reduce adhesion between the dressing and wound surface. Furthermore, for dressing changes, the dressing was soaked with normal saline and then gently lifted up to avoid direct laceration. These measures also helped to minimize pain during the dressing changes.

### Morphometric analysis of wound closure

After various numbers of post-graft weeks (PGWs; 2, 3, and 4 PGWs in Experiment A and 3 and 4 PGWs in Experiment B), all rats were anesthetized by using intraperitoneal injection of sodium pentobarbital (45 mg/kg for Wistar rats and 40 mg/kg for SD rats), the wound was photographed, and the wound area was measured for calculating the wound healing rate (WHR) and wound contraction rate (WCR). In Experiment B, the small amount of autologous skin used for transplantation led to delayed reepithelialization, and the WHR was very low after 2 PGWs. In order to avoid the negative effect of changing the dressing on wound healing, we performed the first dressing change in Experiment B at 3 PGWs. Photographs of each wound with a scaled ruler at its side were taken with a digital camera (Olympus c-5050 zoom, Japan). A new dressing was then applied. The images of the wounds were imported into Scion Image software (Scion Corporation, Frederick, MD, USA) for processing, and planimetry was used to calculate the wound surface area. The original wound area (OWA, 4.0 cm×6.0 cm), the measured whole wound area (MWWA), and the healed wound area (HWA) were used to calculate the WHR and WCR according to the formulas below:
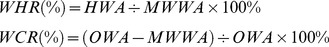



### Histological evaluation of wound healing and measurement of epidermal thickness

**Figure 2:**
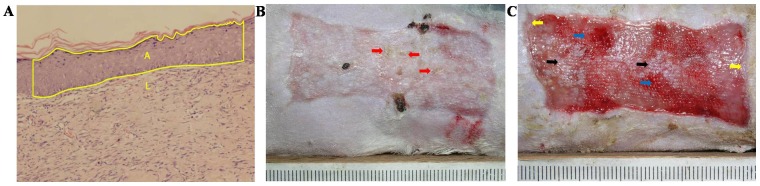
Schematic diagram of measurement of the epidermal thickness and the gross appearance during wound healing. In Figure 2A, L, A, and H represented the length, area, and average thickness, respectively, of the selected epidermal region. H was calculated according to the first mean value theorem for integration: H = A÷L. Figure 2B shows the gross appearance of wound healing in group III in Experiment A at 3 post-graft weeks (PGWs). The red arrows indicate desquamation. Figure 2C shows the gross appearance of wound healing in group I in Experiment A at 2 PGWs. The blue arrows show fresh granulation tissue revealed by the shedding of the allogeneic microskin, the black arrows show the remaining microskin, and the yellow arrows show the de novo epithelium at the wound edge.

To avoid the effect of sample harvest on wound healing, another set of rats (n = 3 in each group at each time point) underwent microskin transplantation as described and were used for histological and immunohistochemical analyses. At various time points post grafting (2, 3, and 4 PGWs in Experiment A and 3 and 4 PGWs in Experiment B), rats were euthanized by administering intraperitoneal injection of sodium pentobarbital (135 mg/kg for Wistar rats and 120 mg/kg for SD rats) and the healed wounds were harvested. As a control, similar-sized skin samples were taken from the backs of normal SD rats. The wound and skin samples were fixed with 4% formaldehyde for 24 hours, embedded in paraffin, and sectioned. After deparaffinization and rehydration, sections were washed thrice with phosphate-buffered saline (PBS) and stained with hematoxylin and eosin (HE). Images were photographed with an IX71 Olympus fluorescence inverted microscope equipped with a DP70 photography system. The length and area of epidermis in the HE-stained sections were measured by using Scion Image software and used to calculate the thickness of the epidermis as shown in Figure 2.

### Immunohistochemical analysis of integrin β1 expression in the healed wound

The expression of integrin β1 after mixed microskin grafting was observed by performing immunohistochemical staining at 2, 3, and 4 PGWs in Experiment A and 3 and 4 PGWs in Experiment B. After deparaffinization and rehydration, sections were treated with a 3% hydrogen peroxide-methanol solution for 10 minutes at room temperature in order to block endogenous peroxidase activity and washed 3 times for 5 minutes each with PBS. The sections were then sequentially incubated with normal goat serum for 40 minutes to block nonspecific binding, with the primary rabbit anti-integrin β1 polyclonal antibody (1∶100, Wuhan Boster Biological Technology LTD, Hubei, China) for 15 hours at 4°C, and with the SABC kit (Wuhan Boster Biological Technology, LTD, Hubei, China) at 37°C to bind the primary antibody. The sections were washed thrice with PBS after each of the above steps. Peroxidase activity was visualized by using a DAB kit (Wuhan Boster Biological Technology, LTD, Hubei, China), resulting in a brown reaction product. Finally, the tissue sections were counterstained with Mayer hematoxylin and coverslipped. Sections of normal SD rat skin were used as positive and negative controls for integrin β1 expression. As a negative staining control, samples were subjected to the staining method above except that the primary antibody was replaced by antibody diluent containing only normal rabbit serum. Images were obtained with an IX71 Olympus fluorescence inverted microscope equipped with a DP70 photography system and imported into Image Pro Plus (version 5.0, Media Cybernetics, Silver Spring, MD, USA) for processing after the method of Chen [7]. Three randomly chosen high power fields (200×) from each sample were imaged to obtain a mean value of each immunostaining parameter for statistical comparison. Staining was defined via the color intensity, and a color mask was made. The mask was then applied equally to all images and the measurements obtained. The intensity of the labeling was determined by using the computer program and assigned a gray value ranging from zero (black) to 256 (white). The immunohistochemical parameters assessed in the detected area included (1) the mean stained area, (2) the mean intensity of the staining, and (3) the mean integrated optical density (mean IOD), mean integral calculus of the mean stained area times the intensity of stain in each pixel in the area indicates the total amount of staining material in that area.

### Statistical analysis

All data were expressed as the mean ± standard deviation (SD). To determine the significance of differences in the WHR, WCR, and integrin β1 expression, variances within the data were analyzed by using Levene's test for analysis of variance. On the basis of the results, Student's *t* test for equal or unequal variance was used to determine the significance of differences between the groups. All tests were two-sided, and a p value of 0.05 or less was considered statistically significant.

## Results

### Effect of mixed microskin transplantation on the wound healing rate in a skin defect wound model

#### Experiment A

**Figure 3:**
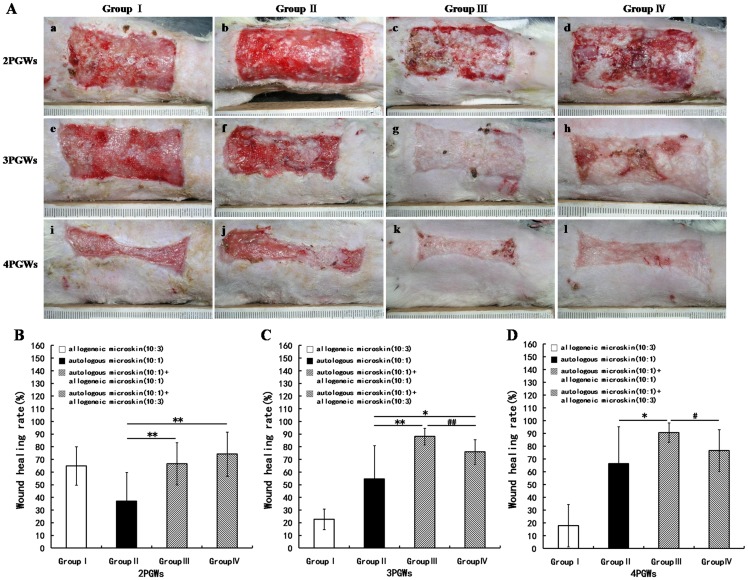
Wound healing at 2, 3, and 4 weeks after mixed microskin transplantation in Experiment A. A shows the gross appearance of the wounds; B, C, and D represent the wound healing rates at 2, 3, and 4 post-graft weeks (PGWs), respectively (* p<0.05, ** p<0.01, # p<0.05, ## p<0.01).

Because of the insufficient amount of autologous skin used in group II, only a small part of the wound was reepithelialized and the rest filled mostly with granulation tissue at 2 PGWs. At the same time point, however, the majority of the wound in groups III and IV was reepithelialized and the rest was filled with granulation tissue on which there were some scattered whitish skin islets. The neoepithelium was soft and fresh. At 3 and 4 PGWs, the reepithelialized wound area had increased somewhat in group II, but reepithelialization was almost complete in groups III and IV, with some scattered scarring and desquamation. Groups III and IV exhibited desquamation rather than obvious shedding of allogeneic microskin. Because no autologous microskin was used in group I, an adverse phenotype was observed in which the entire wound was filled with granulation tissue, with some *de novo* epithelium appearing at the wound edge, accompanied by shedding of the grafted allogeneic microskin from 2 to 4 PGWs (Figure 3A).

The WHR at 2 PGWs was obviously lower in group II than in groups III and IV (Figure 3B). At 3 PGWs, the WHR was still remarkably lower in group II than in groups III and IV, and, more importantly, was higher in group III than in group IV (Figure 3C). The WHR at 4 PGWs was significantly lower in groups II and IV than in group III (Figure 3D).

#### Experiment B

**Figure 4:**
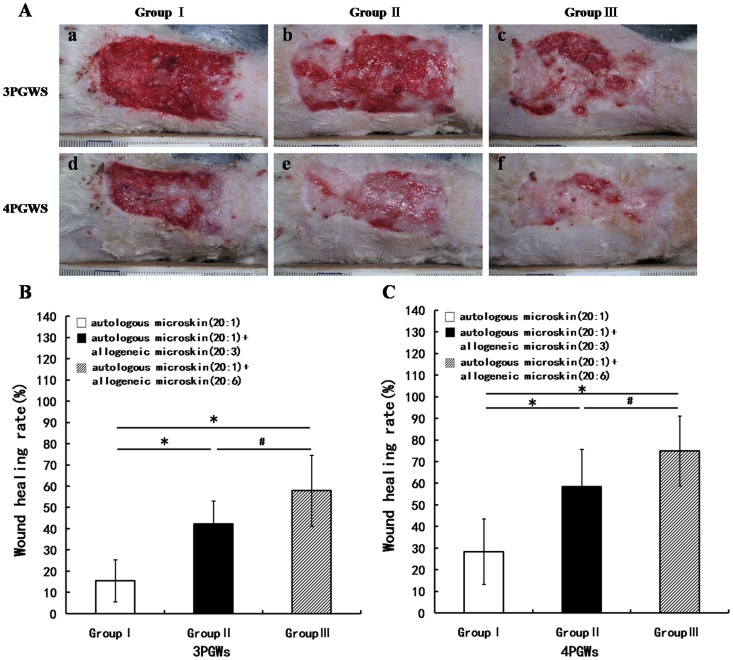
Wound healing at 3 and 4 weeks after mixed microskin transplantation in Experiment B. A shows the gross appearance of the wounds; B and C show the wound healing rates at 3 and 4 post-graft weeks (PGWs), respectively (* p<0.01, # p<0.05).

Much less autologous skin was used in Experiment B than in Experiment A, which delayed wound reepithelialization. However, the addition of allogeneic skin still improved wound healing (Figure 4A). The WHR at 3 PGWs was obviously lower in group I than in groups II and III. More significantly, it was higher in group III than in group II (Figure 4B). At 4 PGWs, the WHR was still remarkably lower in group I than in groups II and III and was higher in group III than in group II (Figure 4C).

### Influence of mixed microskin transplantation on the wound contraction rate in a skin defect wound model

**Figure 5:**
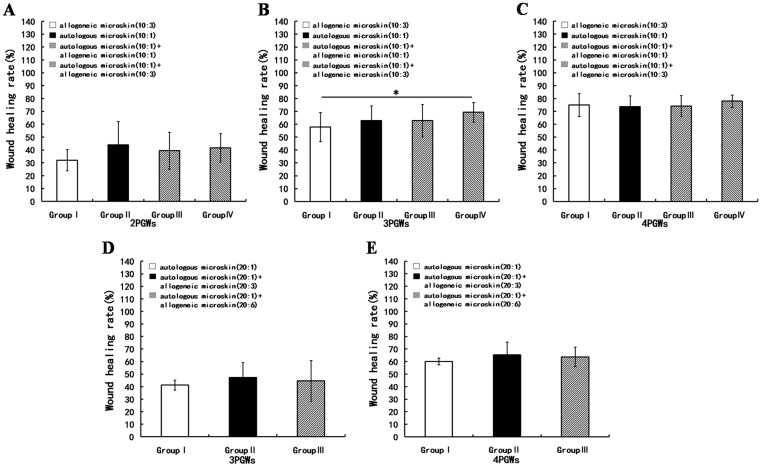
Wound contraction rates after mixed microskin transplantation in Experiments A and B. A, B, and C show the wound contraction rates at 2, 3, and 4 post-graft weeks (PGWs), respectively, in Experiment A (* p<0.05).

The wounds contracted constantly after mixed transplantation in all groups in Experiments A and B (Figure 5). The inter-group differences in WCR were almost never statistically significant except between groups I and IV at 3 PGWs in Experiment A (Figure 5B).

### Histological differences in wound healing and epidermal thickness after mixed microskin transplantation

**Figure 6:**
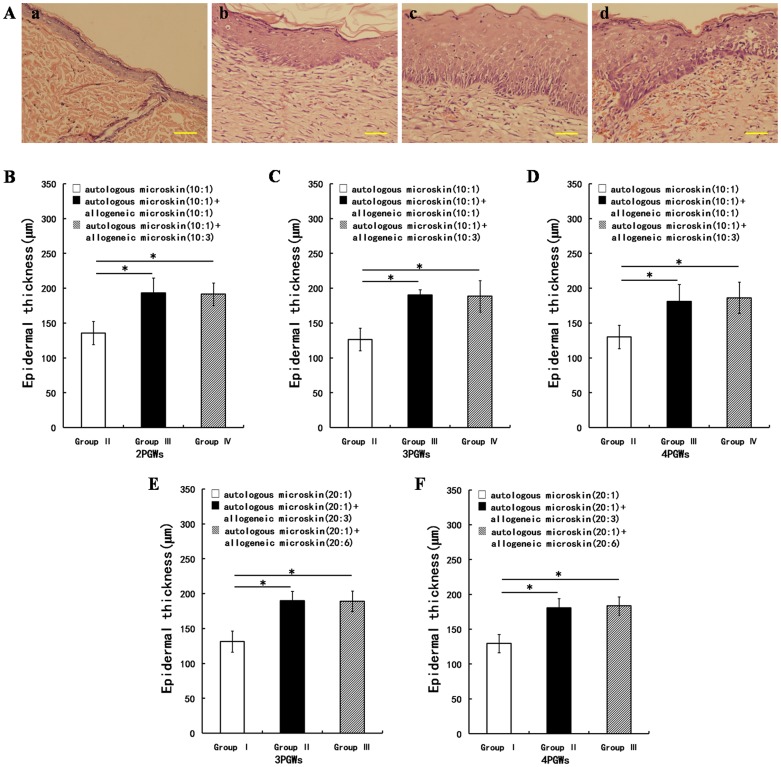
Changes in the histological appearance and epidermal thickness of the healed wounds. Aa shows the normal skin in Sprague-Dawley (SD) rats; Ab, Ac, and Ad show the histological appearance of the healed wounds at 2 post-graft weeks (PGWs) in groups II,III, and IV, respectively, in Experiment A (hematoxylin and eosin; scale bar, 50 µm). B, C, and D show the changes from 2 to 4 PGWs in the epidermal thickness of the healed wounds in 3 groups in Experiment A. E and F show the changes from 3 to 4 PGWs in the epidermal thickness of the healed wounds in 2 groups from Experiment B (µm, 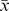
± s), * p<0.05.

In experiment A, the neoepithelium exhibited altered stratification at 2 PGWs in all groups except group I because of the shedding of grafted allogeneic microskin. Although the normal epidermis in the control SD rats consisted of only 3–4 layers of cells (Figure 6 Aa), the neoepithelium in groups II,III, and IV was thickened, with prominent granular and spinous layers (Figure 6 Ab, Ac, and Ad). The majority of the basal cells were columnar in shape (Figure 6 Ac). Mild mononuclear infiltration was observed in the stratum papillare dermidis in group IV (Figure 6 Ad). After mixed microskin transplantation, the interface between the epidermis and dermis was generally almost flat, but shallow rete ridges were present in a few samples. In groups II,III, and IV at 4 PGWs, the basal cells in the epidermis were regularly arrayed and the dermal collagen consisted mainly of mature collagen fibers. Histological examination of wound samples from Experiment B was performed at 3 and 4 PGWs, and the results were similar to those of the corresponding groups in Experiment A.

The epidermal thickness in normal SD rats was 32.72±1.97 µm. The thickness of the epidermis was obviously larger in groups II –IV in Experiment A at 2, 3, and 4 PGWs and in groups I–III in Experiment B at 3 and 4 PGWs than in normal SD rats. In Experiment A, the epidermis was thicker in groups III and IV than in group II, and there was no significant difference between groups III and IV at any time point (Figure 6B, C, and D). The epidermal thickness results in Experiment B were similar to those in Experiment A (Figure 6E and F).

### Immunohistochemical changes in integrin β1 expression in the healed wound after mixed microskin transplantation

**Figure 7:**
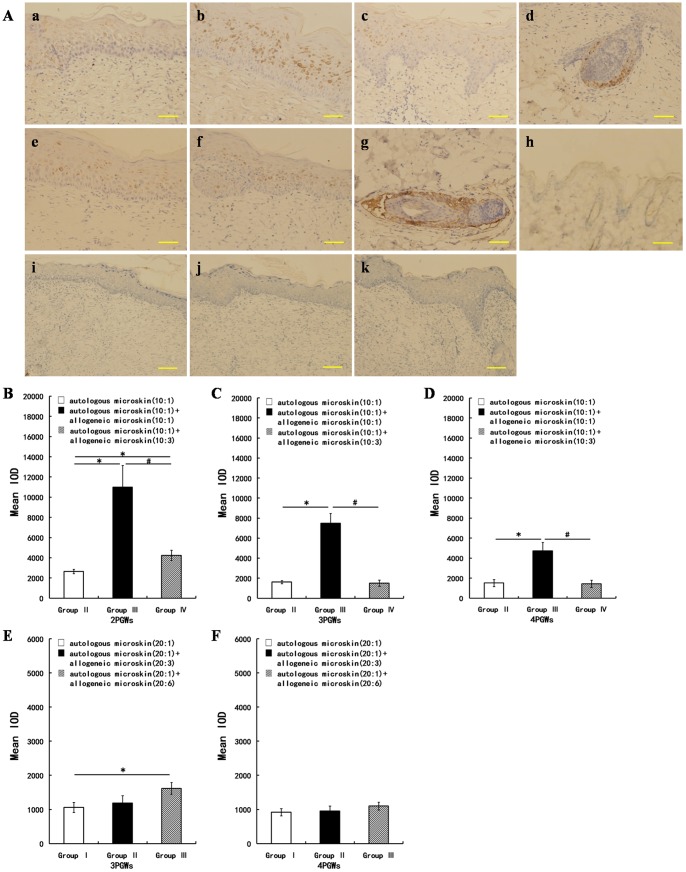
Immunohistochemical detection and quantification of integrin β1 expression in the healed wounds in Experiments A and B. Aa, Ab, and Ac show integrin β1 expression at 2 post-graft weeks (PGWs) in groups II,III, and IV, respectively, in Experiment A. Ad shows integrin β1 expression in the hair follicle in group IV in Experiment A. Ae and Af show integrin β1 expression at 2 PGWs in groups II and III, respectively, in Experiment B. Ag and Ah show expression of integrin β1 in the hair follicle and no expression of integrin β1 in the epidermis, respectively, of normal Sprague-Dawley (SD) rat skin. Ai, Aj, and Ak show negative control staining of 2 PGW sections from groups II, III, and IV, respectively, in Experiment A. Scale bar, 50 µm in Aa–Ah and 100 µm in Ai–Ak. In Experiment A, the mean integrated optical density (IOD) of integrin β1 expression at 2 PGWs was higher in groups III and IV than in group II (* p<0.05), and, more interestingly, was highest in group III among these 3 groups (* p<0.05, # p<0.05). At 3 and 4 PGWs, the integrin β1 expression remained higher in group III than in groups II and IV (* p<0.05, # p<0.05). In Experiment B, the integrin β1 expression at 2 PGWs was stronger in group III than in group I (* p<0.05), and no intergroup difference was found at 4 PGWs.

There was no expression of integrin β1 in the epidermis of the normal control SD rats. All of the wound biopsies from groups II,III, and IV in Experiment A from 2 to 4 PGWs and those from groups II and III in Experiment B at 3 and 4 PGWs showed positive staining for integrin β1 in the suprabasal layers, especially in the spinous and granular layers. Expression was also seen in hair follicles (Figure 7Ad). According to the mean IOD value in Experiment A, the epidermal expression of integrin β1 was significantly greater in group III than in groups II and IV at 2, 3, and 4 PGWs. In addition, integrin β1 expression at 2 PGWs was remarkably stronger in group IV than in group II (Figure 7B, C, and D). Nevertheless, in Experiment B, although integrin β1 expression at 2 PGWs was stronger in group III than in group I, no significant intergroup difference was found at 4 PGWs (Figure 7E and F).

## Discussion

In patients with extensive skin destruction, large open wounds cannot be repaired promptly because of the limited amount of autologous skin and consequently become the main source of infection and other complications. Many researchers and surgeons have attempted for more than 3 decades to find efficient engineered skin substitutes to address this problem [3], [8]. However, few of their solutions have been used widely in clinical practice [9]. Another approach has been to develop novel skin grafting techniques, such as intermingled skin grafting and autologous microskin grafting [1], [2]. These 2 skin grafting methods are widely used in China for the treatment of extensive deep burns.

Intermingled skin grafting uses small autologous skin islets (usually 0.5 cm×0.5 cm) inlaid in a large sheet of allogeneic or xenogeneic skin. The inlaid autologous skin islets increase the local level of IL-10 and thus induce local immune tolerance; this is called the autologous skin islet effect (ASIE) [4] and delays the subsequent rejection of the allogeneic or xenogeneic skin graft [1]. The prominent advantage of autologous microskin grafting is that an even smaller amount of autologous skin is used to make tiny microskin particles (less than 1 mm^3^), and the cells at the edges of the microskin particles can migrate and proliferate in all directions [2]. The aim of this study on mixed autologous and allogeneic microskin grafting was to integrate these 2 skin grafting techniques while preserving the advantages of both in order to achieve better wound healing while using less autologous skin and avoiding obvious rejection.

### Mixed microskin transplantation substantially improves the wound healing rate

Experiment A showed that the WHR at 2 and 3 PGWs was considerably higher in groups III and IV than in group II and that the WHR at 3 and 4 PGWs was even higher in group III than in groups II and IV. Experiment B produced similar results. We can therefore conclude that mixed transplantation of autologous and allogeneic microskin can promote wound healing.

We speculate that this phenomenon might largely be attributable to the following factors. First, many of the cells from the transplanted allogeneic microskin, such as keratinocytes and fibroblasts, are important sources of all kinds of cytokines and growth factors that may promote wound healing [10]. Second, epidermal-mesenchymal communication is critical for exchanging information between keratinocytes and fibroblasts in skin morphogenesis during development and probably also in the maintenance of the integumentary structure of adult skin [11]. Keratinocyte-fibroblast interactions play significant roles in the wound healing process [12]. It has been shown that during the reepithelialization phase of wound healing, keratinocytes depend on signals from dermal fibroblasts to reestablish a functional epidermis [12], [13]. Nowinski et al. [14] demonstrated in a keratinocyte-fibroblast co-culture system that a large number of genes coding for growth factors, cytokines, and their receptors were regulated in fibroblasts by keratinocyte-derived factors. In the present study, autologous and allogeneic microskin particles were thoroughly mixed to allow direct contact between keratinocytes and fibroblasts without the partition imposed by the basement membrane.

### The ratio of autologous to allogeneic microskin in the mixture impacts wound healing

When the amount of autologous microskin is fixed, the addition of more allogeneic microskin to a wound might improve wound healing by providing more growth factors and keratinocyte-fibroblast interactions [15]. Even acellular dermal matrix can also facilitate the regeneration of mature dermis in a rat skin wound [16]. However, the use of a greater amount of allogeneic microskin usually exacerbates the host versus graft response, which might have a negative effect on wound healing [17]. Nevertheless, if more of the wound area is covered by allogeneic microskin, less of it must be covered by keratinocyte migration and proliferation from autologous microskin. Therefore, allogeneic microskin could have opposing effects on wound healing.

Notably, in Experiment A, when the area expansion ratio of autologous microskin was 10∶1, allogeneic microskin produced better wound repair when added at an area expansion ratio of 10∶1 than when added at 20∶1. However, although mixed microskin grafting still accelerated wound healing in Experiment B, the WHR was less satisfactory than in Experiment A because only half the amount of autologous skin was used. When using this extremely limited amount of autologous skin for transplantation, improved wound healing could be achieved by the addition of a greater amount of allogeneic microskin. These results indicate that the ratio of autologous to allogeneic microskin in the mixture also has an important effect on wound healing.

### Increased ectopic expression of integrin β1 in the epidermis may play pivotal roles in improving wound healing after mixed transplantation

Integrins are the main cell surface receptors for proteins within the extracellular matrix (ECM) and contribute to development [18], [19], hemostasis [20], and the immune response [21]. They are heterodimeric transmembrane proteins consisting of an α and a β subunit that play crucial roles in cell-cell and cell-matrix interactions. Each consists of a large extracellular domain, a single transmembrane segment, and a relatively short cytoplasmic tail [22]. These subunits underpin cell adhesion and migration and are also involved in cell proliferation, programmed cell death, and differentiation [23]. The major integrins in the intact epidermis are α2β1, α3β1, and α9β1, which bind various extracellular matrix proteins such as laminins, collagen I, tenascin C and fibronectin, and α6β4, an integral component of hemidesmosomes that binds laminin 5 [24], [25], [26]. The characterization of integrin β1-deficient mice has revealed crucial roles for the β1 integrin subfamily in hair follicle development [27], skin integrity [28], and cutaneous wound repair [29]. More importantly, the integrin β1 subunit has been found to be a marker of keratinocyte stem cells (KSCs) [30], [31] and is the usual means of identifying such cells. To explore the primary mechanism by which mixed microskin transplantation improves wound healing, we investigated the expression patterns of integrin β1, epidermal growth factor (EGF), and epidermal growth factor receptor (EGFR), as well as the epidermal thickness, in the healed wound after mixed transplantation.

In the skin of normal rodents, epidermal stem cells are largely localized to the area of the hair follicle bulge. To date, there has been no report of the distribution of KSCs and the characteristics of their marker expression after mixed transplantation. In the present study, we found no expression of integrin β1 in the epidermis of normal SD rats. Intriguingly, however, ectopic integrin β1 expression was observed in groups II –IV in Experiment A and in groups I–III in Experiment B. Cells expressing integrin β1 were scattered in the suprabasal layers, especially in the spinous cell layer and the granular layer. More importantly, we found that in Experiment A, integrin β1 expression from 2 to 4 PGWs was higher in group III than in the other groups, consistent with the better wound healing rate and more thickened epidermis in those groups. In Experiment B, group III also showed an encouragingly higher wound healing rate concomitant with higher integrin β1 expression and a thicker epidermis at 3 PGWs. Integrin β1 is normally a cell surface protein. However, we observed intracellular expression of integrin β1 in the present study. We speculate that this phenomenon may be attributable to the following processes. (1) New synthesis of integrins: Hotchin NA et al. [32] found that intracellular transport of newly synthesized integrins in human epidermal keratinocytes was inhibited by terminal differentiation. (2) Integrin internalization: this process occurs through clathrin-dependent [33] and clathrin-independent [34] (via caveolae or macropinocytosis) mechanisms, and many integrins can enter the cell via more than one route. Motifs in the cytoplasmic tails of integrins are crucial to the regulation of integrin endocytosis and recycling via a number of pathways [35]. These results imply that epidermal keratinocytes with ectopic expression of integrin β1 may play pivotal roles in improving wound healing after mixed transplantation.

Zhang et al. [36] found markedly higher expression of integrin β1 in dedifferentiation-derived cells than in control cells, and the characteristics of these dedifferentiation-derived cells, which were capable of regenerating a skin equivalent, resembled those of KSCs. Furthermore, when leg ulcers were treated topically with recombinant human EGF, some stem cells or stem cell-like cells appeared in the spinous and granular layers of the regenerated epidermis. These undifferentiated cells may have been derived from dedifferentiation of the differentiated epidermal cells [37]. Although the present study found no intergroup difference in EGF or EGFR expression (data not shown), which might be because the expression of EGF and EGFR peaked within 2 PGWs, we still believe that many secreted factors, including growth factors, cytokines, and components of the extracellular matrix, act together to facilitate wound healing by inducing ectopic expression of integrin β1 in the epidermis.

In conclusion, mixed transplantation of autologous and allogeneic microskin grafts in appropriate proportions shows an encouraging effect on wound healing while reducing the amount of autologous skin used. The addition of allogeneic microskin at an appropriate ratio and ectopic expression of integrin β1 in epidermal keratinocytes contribute to this improvement in wound healing. However, future studies are needed to determine what the crucial cells and molecules are, where they come from, and how they affect wound healing.
